# The Objects and Methods of Medical Education

**Published:** 1909-09-04

**Authors:** 


### The Hospital

A Journal of

The Medical Sciences and Hospital Administration.

New Series. No. 132, Vol. V. [No. 1204, Vol. XLVI.] SATURDAY, SEPTEMBER 4, 1909.

### EDUCATIONAL NUMBER.

### THE OBJECTS AND METHODS OF MEDICAL EDUCATION.

It is easy to define in general terms the objects of medical education at the present day. They are, of course, to turn out a class of cultured gentlemen, as well qualified to prevent and cure diseases as the knowledge of the times makes it possible for them to be. The proposition, it will be admitted, is beautifully large and vague, and there is some reason to fear that the methods at present in vogue are, if anything, vaguer than the proposition. It is not therefore inappropriate, especially at this season, when a new generation of medical scholars is about to take up its task, to formulate more precisely the ideals at which medical education should be aimed, with a view to permitting a comparison between what we have and what we ought to wish for.

It is a premiss of vital moment that any scheme of education designed for a class, and not for an individual, should be drawn to the scale of an average intellectual capacity. Otherwise idealism is purchased at the expense of practicability; and, though the products of such a scheme may include here and there a practitioner of phenomenal brilliancy, the general average will be low; since the commonplace individual, finding himself incapable of digesting the over-ample intellectual fare presented to him, is likely to give up the unequal struggle and sink into sloth or despondency. We will assume therefore that the function of a scheme of medical education is to produce general practitioners in the highest possible state of professional efficiency, a condition involving far more than the possession of mere technical knowledge in abundance. The ambitious man, and he who evinces a leaning in some special direction, may safely be left to their own devices once they have received the training necessary to equip them as high-class general practitioners. It is with the average that we are concerned, and our object is to make the average high, not by virtue of an isolated marvel or two, but by virtue of a general levelling-up.

Admitting this, we must at once dismiss from our minds all hope of making the practitioner of the future an encyclopædia of science. This has been attempted in the past, and not unnaturally. There is hardly a branch of natural science with which Medicine, in the large sense, does not at some point come into touch, and it is not unreasonable to affirm that the more comprehensive the horizon of science commanded by a doctor, the more balanced, sane, and perspicacious will be his judgment of the particular problems he must encounter in his daily practice. But the hope is not tenable. The body is a chemical factory unapproached in the intricacy of its operations; there is not a function of it which does not involve a chemical interchange, and it is probable that there is not a distemper of it, excepting mechanical injury, which is not based on a similar foundation. Therefore, one might argue with some show of reason, the prime essential of medical education is chemistry, including of course bio-chemistry. Yet the proposition has only to be thus baldly stated for the fallacy of it to become apparent. The fact is that there is scarcely a branch of knowledge which may not on occasion be directly useful to a doctor. But since art is long and life short, what the novice requires is that a rigid selection should be made for him of those items, and those alone, which are intimately related to medical efficiency.

There is no better way of arriving at the proper direction of medical education than to analyse the generic qualities and characteristics of general practitioners who have proved themselves successful. For, putting aside the rare instances in which success has been won by unblushing effrontery and unrestrained abuse of truth, it is correct to say that no man succeeds in general practice who has not the capacity to benefit his patients; and he who fails in this has failed to justify his professional existence, be his academic merits what they may. Now it is a matter of common experience that academic merit and professional success do not by any means necessarily go hand in hand. Many a man whose technical knowledge is minimal achieves pronounced success because what we may call his technical wisdom is great; while, conversely, everyone is acquainted with doctors whose high technical knowledge does not protect them from relative failure. We are driven then to the conclusion that technical knowledge is not the end-all of medical education, and we may spare a moment to inquire somewhat into the essence of this technical wisdom.

Technical knowledge—knowledge of a craft—implies an acquaintance with the instruments of a craft; but technical wisdom as we wish it to be understood implies a faculty of judgment as to the proper instrument to be used in any particular conjunction of circumstances. In the case of a handicraft, such as carpentering, technical knowledge and technical wisdom are not likely to be far divorced, since the material to be worked upon has but limited attributes, and these well-ascertained and constant, while the relation between the instrument to be used and the purpose to be achieved can be reduced to a simple rule. But the doctor's material—namely, human nature, is unstable in the last degree. Not only does every individual react differently from his neighbours to a given stimulus, but the individual's reaction to an identical stimulus is capable of variation from day to day. Moreover, there is no simple rule by which a doctor may know which of the many instruments of his trade is the suitable one for any given case. For example: A patient presents the symptoms of gastric dyspepsia. Now technical knowledge tells the doctor that these symptoms may be combated by bismuth, or bitters, or aperients, or gastro-enterostomy, or dieting; but only technical wisdom will lead him to perceive that the root of the whole mischief is anxiety, and that the proper instrument to be used is not a drug, nor a knife, but comfortable conversation and consoling advice.

It seems then that above all things a medical student must be taught to develop acuity and breadth of mental vision, and a faculty for analysing human nature, in order that he may be prepared to detect, and meet, latent characteristics knowledge of which is vital to his success in treatment, but of which humanity in general, and particularly ailing humanity, is often profoundly secretive. In other words he must be taught to cultivate tact, the quality which enables a man to peer into his neighbour's mind, to measure his temperament, to anticipate the effect of events upon that temperament, even to reconstruct from observed effects the causes which have been operative in their production, and finally to bring to bear the curative measures, whether material or immaterial or both, which his technical wisdom pronounces to be most appropriate. It may be urged firstly that this is not medical education, and secondly that it cannot be taught. The answer is that what appears to be the prime factor of success in a profession can hardly be considered foreign to the schedule of instruction in that profession. As to the second point, it is true that to some people the exercise of tact is natural, while others are incapable of compassing it—much as some folks of average intelligence are incapable of assimilating the most elementary notions of mathematics. Nevertheless there is little doubt that, were the matter insisted upon by teachers in the schools, students would go out into the world with a much juster appreciation of the curative virtues of human-kindness. We do not wish to imply that our teachers (or students for that matter) are actively harsh. Far from it. But they are not as a rule actively sympathetic. Pre-occupied with the scientific, they either neglect, or at least do not emphasise, the human side of medicine, an appreciation of which forms the chief therapeutic agent of many a successful general practitioner.

But although we have insisted with so much urgency upon the non-material aspect of medical education, we recognise well enough that no exercise of tact or penetration will control a post-partum hæmorrhage, relieve intestinal obstruction, or restore a failing heart. It is with the provision of such knowledge that medical education, on its medical, as opposed to its merely scientific, side, has up to the present exclusively concerned itself; and, though we have advanced reasons for supposing that this monopoly of attention is not deserved, the subject is of immense importance. The problem here, though simpler than the other in that it is more defined, is yet an enormous one. It is to provide a man, within a period of five years, with sufficient technical knowledge to make him an efficient physician, surgeon and obstetrician. Now these are immense subjects, and there are certain others so intimately related with them as to form almost a part of them—namely, Anatomy, Physiology, Bacteriology, and Pathology. No amount of pruning can legitimately reduce this list; but there is quite a number of subjects, certainly cognate to medicine in some particulars, yet sufficiently remote to make them relatively undeserving. These are the subjects upon which it is usual to spend the first two years of the medical curriculum—namely, Biology, Physics, Chemistry, and Botany. There is a considerable body of opinion that these subjects are not entitled to a place in the strictly medical curriculum. Not that they are unimportant (since knowledge of them, especially of physics and chemistry, is often of notable service to the practitioner), but because in the first place there are matters of higher moment which call for attention, and in the second that the amount of time at present devoted to these subjects is insufficient to give the student more than a smattering of them, a knowledge too superficial to be turned to good account. Even with regard to Pathology (which is, in practice, to a large extent synonymous with Morbid Anatomy), there is room for doubt whether the tendency of the times is not to make too much of it; for long familiarity with the morbid anatomy of diseases which have proved fatal tends to produce a pessimistic attitude in a doctor, an attitude pernicious both to himself and his patients, and not justified by the generality of medical experience. In brief, then, we may say that the studies of a medical student during the five years of his curriculum should be limited to those immediately concerned with the prevention or cure of disease. The present preliminary subjects should be made truly preliminary to the study of medicine, instead of trespassers. They are subjects eminently fitted to be learnt during the later years of school life, while five years is none to long to master what is required of the efficient practitioner. The first desideratum for the person who is to spend his life in dealing with sick people is the study of sick people under the direction of experienced teachers, and the whole of the curriculum should be devoted to it. Should such a scheme become operative we should not, as we do at present, see large and vastly important branches of disease, such as the specific infectious fevers and insanity, cavalierly dealt with in a dozen or a score of flying visits, snatched at the expense of other studies. The great requirements of medical education on its material side are that it should be as far as possible clinical, and that those who order it should allot to each of its elements time strictly proportional to the value of that element in building up an efficient physician.

